# A comprehensive assessment of fungal communities in various habitats from an ice-free area of maritime Antarctica: diversity, distribution, and ecological trait

**DOI:** 10.1186/s40793-022-00450-0

**Published:** 2022-11-15

**Authors:** Tao Zhang, Dong Yan, Zhongqiang Ji, Xiufei Chen, Liyan Yu

**Affiliations:** 1 China Pharmaceutical Culture Collection, Institute of Medicinal Biotechnology, Chinese Academy of Medical Sciences & Peking Union Medical College, Beijing, People’s Republic of China; 2 Xinxiang Key Laboratory of Pathogenic Biology, Department of Pathogenic Biology, School of Basic Medical Sciences, Xinxiang Medical University, Xinxiang, 453003 Henan People’s Republic of China; 3 Key Laboratory of Marine Ecosystem Dynamics, Second Institute of Oceanography, Ministry of Natural Resources, Hangzhou, People’s Republic of China

## Abstract

**Background:**

In the ice-free area of maritime Antarctica, fungi are the essential functioning group in terrestrial and marine ecosystems. Until now, no study has been conducted to comprehensively assess fungal communities in various habitats in Antarctica. We aimed to characterize fungal communities in the eleven habitats (i.e., soil, seawater, vascular plant, dung, moss, marine alga, lichen, green alga, freshwater, feather) in the Fildes Region (maritime Antarctica) using next-generation sequencing.

**Results:**

A total of 12 known phyla, 37 known classes, 85 known orders, 164 known families, 313 known genera, and 320 known species were detected. Habitat specificity rather than habitat overlap determined the composition of fungal communities, suggesting that, although fungal communities were connected by dispersal at the local scale, the environmental filter is a key factor driving fungal assemblages in the ice-free Antarctica. Furthermore, 20 fungal guilds and 6 growth forms were detected. Many significant differences in the functional guild (e.g., lichenized, algal parasite, litter saprotroph) and growth form (e.g., yeast, filamentous mycelium, thallus photosynthetic) existed among different habitat types.

**Conclusion:**

The present study reveals the high diversity of fungal communities in the eleven ice-free Antarctic habitats and elucidates the ecological traits of fungal communities in this unique ice-free area of maritime Antarctica. The findings will help advance our understanding of fungal diversity and their ecological roles with respect to habitats on a neighbourhood scale in the ice-free area of maritime Antarctica.

**Supplementary Information:**

The online version contains supplementary material available at 10.1186/s40793-022-00450-0.

## Background

As a result of climate change, water availability, CO_2_, temperature, and UV levels have been changing in Antarctica [1]. Notably, ice-free areas currently cover less than one percent of Antarctica [2] and could expand by close to 25 percent by 2100 [3]. Many ice-free areas have emerged from the retreating ice in marine Antarctica, and glacial erosion is the dominant land-forming factor. As ice-free areas are home to Antarctic biodiversity (e.g., microbes, vascular plants, lichens, mosses, algae), the increase in ice-free areas could drastically change the availability and connectivity of biodiversity habitats (e.g., uncover potential new habitats for species [3]). Some non-native species have been introduced to Antarctic ice-free environments by natural dispersal or human activities (e.g., marine invertebrates, inserts, and plants) [4]. In addition, climate warming could influence the composition of microbial communities in Antarctica. Previous studies have shown that warming leads to significant changes in soil fungal abundance in Antarctica [5, 6].

To date, over 1000 non-lichenized fungal species [7] and 500 lichenized fungal species [8] have been recorded by collection or isolation from Antarctica. In recent years, many studies have used the next-generation sequencing (NGS) technique to reveal fungal diversity in various habitats of Antarctica, such as soil [6, 9–14], sediment [15], vegetation [14, 16], air [17], and freshwater [14, 18]. These molecular surveys suggest that the true fungal diversity may be far greater than that has been recorded. Besides, there are no studies that consider various habitats (e.g., soil, lichen, vascular plant, moss, freshwater, seawater, dung, air, feather, green alga, and marine alga) as a whole. In terms of microbes, it was believed that "everything is everywhere, but the environment selects" (the Baas-Becking’s hypothesis [19]). This hypothesis states the joint effect of dispersal capabilities (i.e., spores that aid in dispersal and propagation) and environmental selection. It is still unclear to what extent habitat specificity and habitat overlap determine fungal assemblages in a local ice-free area.

In both terrestrial and marine ecosystems, fungi typically live in highly diverse communities and serve a variety of ecological functions, such as saprotrophs (living on dung, leaf, plant, soil, wood), symbiotrophs (participating in mutualistic symbioses: ectomycorrhizal, ericoid mycorrhizal, endophyte, lichenized), and pathogens of plants and animals [20]. However, there is only fragmentary information on fungal ecological traits in the soil habitat of maritime Antarctica [13, 21–23]. Until now, the knowledge gap between fungal diversity and their ecological roles is still significant in other habitats of maritime Antarctica.

The present study aims to use the next-generation sequencing (NGS) approach to reveal: (1) the taxonomic diversity of fungal communities in an ice-free area of maritime Antarctica; (2) the extent of habitat specificity and habitat overlap in determining fungal assemblages in various habitat types, including soil, lichen, vascular plant, moss, freshwater, seawater, dung, air, feather, green alga, and marine alga; (3) the ecological traits of fungal communities in various habitats of maritime Antarctica. We hypothesized that environmental selection determined the taxonomic and functional compositions of fungal communities in various habitats from an ice-free area of maritime Antarctica. The findings will improve our understanding of the fungal diversity with respect to environments on a neighbourhood scale, and aid further analysis of fungal ecological roles in this unique ice-free area of maritime Antarctica.

## Materials and methods

### Sample collection

**Fig. 1:**
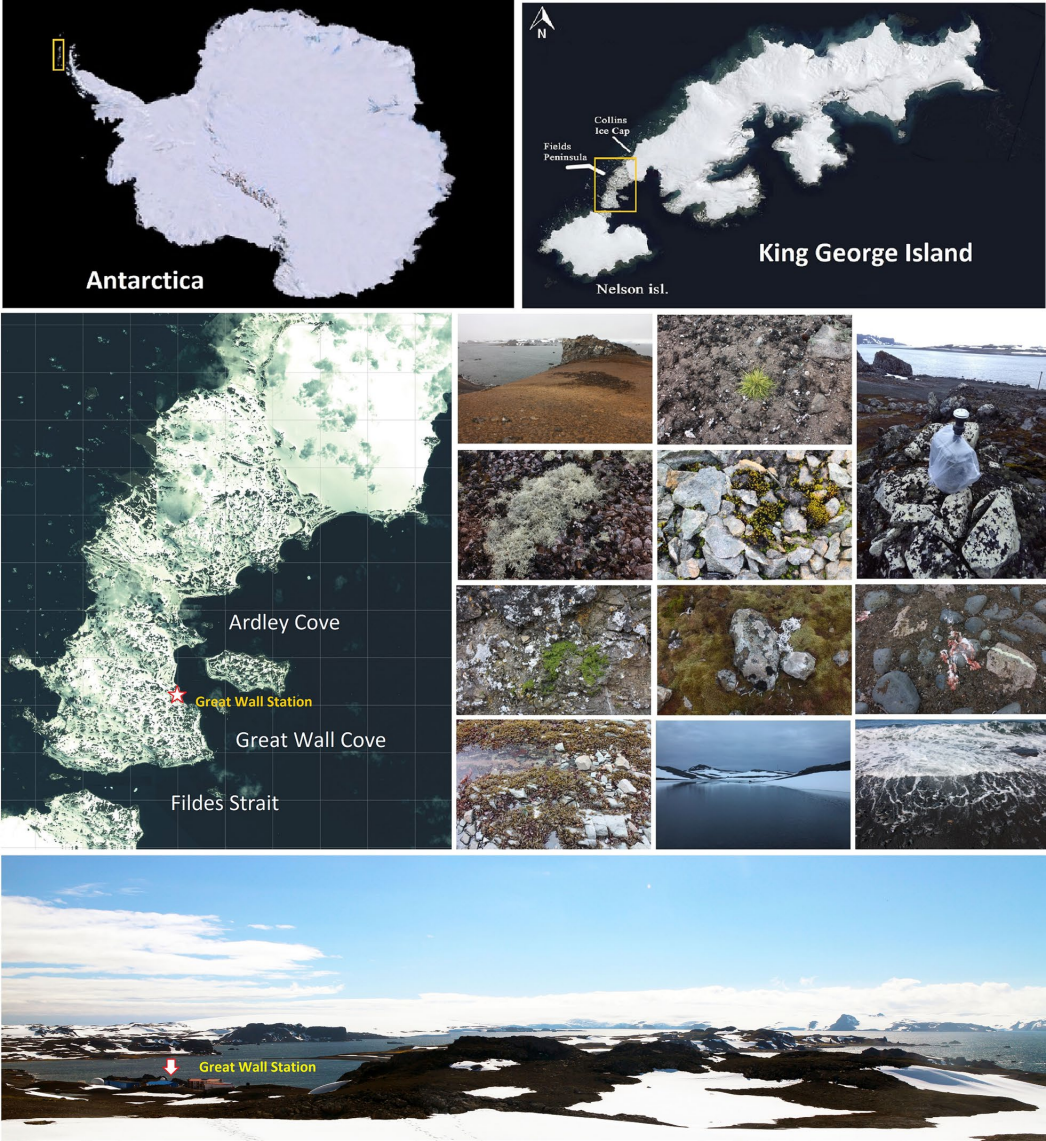
The location of the sampling site and views of eleven habitats (i.e., soil, freshwater, seawater, air, lichen, vascular plant, moss, green alga, marine alga, dung, and feather) in the Fildes Region (maritime Antarctica)

The sampling site is the Fildes Region (King George Island, maritime Antarctica), which is consisted of Fildes Peninsula, Ardley Island, and the northern part of Nelson Island. It is one of the largest ice-free areas in maritime Antarctica and has a relatively high level of biodiversity. The mean annual temperature in this region is − 2.2 °C [24] and has increased on average by 0.7 °C between 1969 and 2013 [25]. In January 2017, a total of 213 samples were collected during the 33rd Chinese National Antarctic Research Expedition (CHINARE-Antarctic) (Fig. 1, Additional file 1: Table S1), including 136 samples in this study (i.e., soil, lichen, vascular plant, freshwater, seawater, dung, air, feather, green alga, and marine alga) and 77 samples in the previous studies (i.e., soil, freshwater, moss, vascular plant) [12, 14].

### DNA extraction and sequencing

(1) For samples of soil and dung, DNA extraction was conducted from about 0.25 g aliquot using MoBio PowerSoil DNA isolation kits (MoBio Laboratories Inc., USA). (2) For samples of freshwater and seawater, 1000 ml water was filtered through 0.2 μm-pore-size membranes (Pall Corporation, USA). Total DNA in membranes was extracted using the PowerWater DNA Isolation Kit (MO BIO Laboratories Inc., USA). (3) For samples of vascular plant, lichen, green alga, and marine alga, before DNA extraction, tissues were surface sterilized and crushed as described by Zhang et al. [26]. DNA extraction was performed using a PowerSoil DNA Isolation Kit (MO BIO Laboratories Inc., USA). (4) For bird feather, the feather was cut into small segments with sterilized scissors and then used a SuperFastPrep-1 Instrument (MP Biomedicals Co., USA) to crush segments. DNA extraction was conducted from segments using a PowerSoil DNA Isolation Kit (MO BIO Laboratories Inc., USA). (5) For air samples, total suspended particles of air were collected using portable ambient air samplers (Air Metrics, USA) as described by Yan et al. [27]. DNA extraction was conducted from the membranes of air samples using a PowerSoil DNA Isolation Kit (MO BIO Laboratories Inc., USA).

The obtained DNA was used for subsequent PCR and sequencing. The fungal nuclear ribosomal internal transcribed spacer 1 (ITS1, approximately 285 bp) was amplified using primer sets that were added a 10-nucleotide barcode to ITS1F [28] and ITS2 [29]. PCR was performed as previously described [12]. The PCR products of the ITS region were extracted from 2% agarose gels and purified using an AxyPrep DNA Gel Extraction Kit (Axygen Biosciences, USA) according to the manufacturer’s instructions and were quantified using QuantiFluor-ST (Promega, USA). Equimolar volumes of purified amplicons were pooled and were paired-end sequenced (2 × 300 bp) on an Illumina MiSeq platform at Majorbio Company (Shanghai, China). Both DNA extraction and PCR were applied to negative control samples. These negative controls did not undergo any more analysis because no quantifiable DNA was found in them. Sequencing was conducted using an Illumina MiSeq platform at Majorbio Company (Shanghai, China).

### Sequencing data treatment

Paired-end reads were merged using FLASH software [30] and assigned to each sample according to the unique barcodes. The raw demultiplexed sequences were processed in QIIME 2 v2022.01 [31]. Paired-end reads were denoised, dereplicated, and filtered for chimeras using the DADA2 plugin [32], as implemented in QIIME 2. Raw reads were trimmed to include only bases with quality scores > 35. The first 26 and 26 nucleotides of the 5′ end of the forward and reverse sequences, respectively, also were trimmed. The 3′ ends of the forward and reverse sequences were truncated at positions 230 and 220, respectively. The number of sequences used to train the error model was set to 100,000. De novo clustering using a threshold of 100% of similarity was performed using VSEARCH [33], as implemented in QIIME 2. Taxonomic assignments were determined for amplicon sequence variants (ASVs) using classify-sklearn with a naïve Bayes classifer [34] against UNITE Fungi v8.3 reference database [35] pre-trained to ITS1. ASVs with an abundance of less than 5 sequences were removed. Samples were then subsampled to 20,180 sequences per sample. In addition, MAFFT [36] was used to align with representative sequences of ASVs (Additional file 2: Table S2) (FFT-NS-1 method), and a phylogenetic tree was constructed using Average linkage (UPGMA) method.

### Statistical analyses

Statistical analysis was carried out on the MicrobiomeAnalyst (marker data profiling, MDP) [37]. The fungal community compositions in the samples were ordinated using Principal Coordinates Analysis (PCoA) with the unweighted UniFrac distance method. Dendrogram analysis (clustering algorithm: Ward; distance measure: unweighted UniFrac distance) of fungal communities in the samples of eleven habitats was also performed to explore their relationships. Analysis of similarity (unweighted UniFrac distances; 999 permutations) was used to validate the dissimilarity of fungal communities among different habitat types. A Venn diagram showing the number of fungal ASVs in eleven habitats was constructed online (www.omicshare.com/tools/Home/Soft/venn). A correlation network analysis was conducted to explore interactions of fungal genera based on Spearman’s rank correlation test (Permutation: 100, *p*-value threshold: 0.01, correlation threshold: 0.75). A linear discriminant analysis effect size (LEfSe) analysis was used to explore the significantly different fungal taxonomic groups (i.e., phylum, class, order, family, and genus) among the eleven habitat types based on the factorial Kruskal–Wallis test. The ecological traits of fungal communities were determined using FungalTraits [38]. Potentially pathogenic fungal species of interest were determined according to the Atlas of Clinical Fungi [39].

## Results

### Characteristics of fungal community compositions in the eleven habitats

A total of 9,864,604 raw reads from 213 samples were obtained. The number of raw reads ranged from 30,400 to 139,071 per sample. After being denoised, dereplicated, and filtered for chimeras, the number of reads was reduced to 8,040,848 for a total number of 17,236 ASVs. The number of trimmed reads ranged from 7 to 125,173 per sample (Additional file 3: Table S3). The read number was reduced to 8,035,065 for 15,382 ASVs in 213 samples following the elimination of rare ASVs (less than five reads). Each sequence library was then rarefied to 20,812 reads which retained 4,204,024 reads in 202 samples and 14,814 ASVs. The ASV number detected in each sample was in the range of 2 to 502 (Additional file 4: Table S4). The ASVs could be identified at different taxonomic levels of precision: 9,892 ASVs were assigned to unknown phyla, whereas 4,922 ASVs were assigned to 12 known phyla, 37 known classes, 85 known orders, 164 known families, 313 known genera, and 320 known species.

**Fig. 2:**
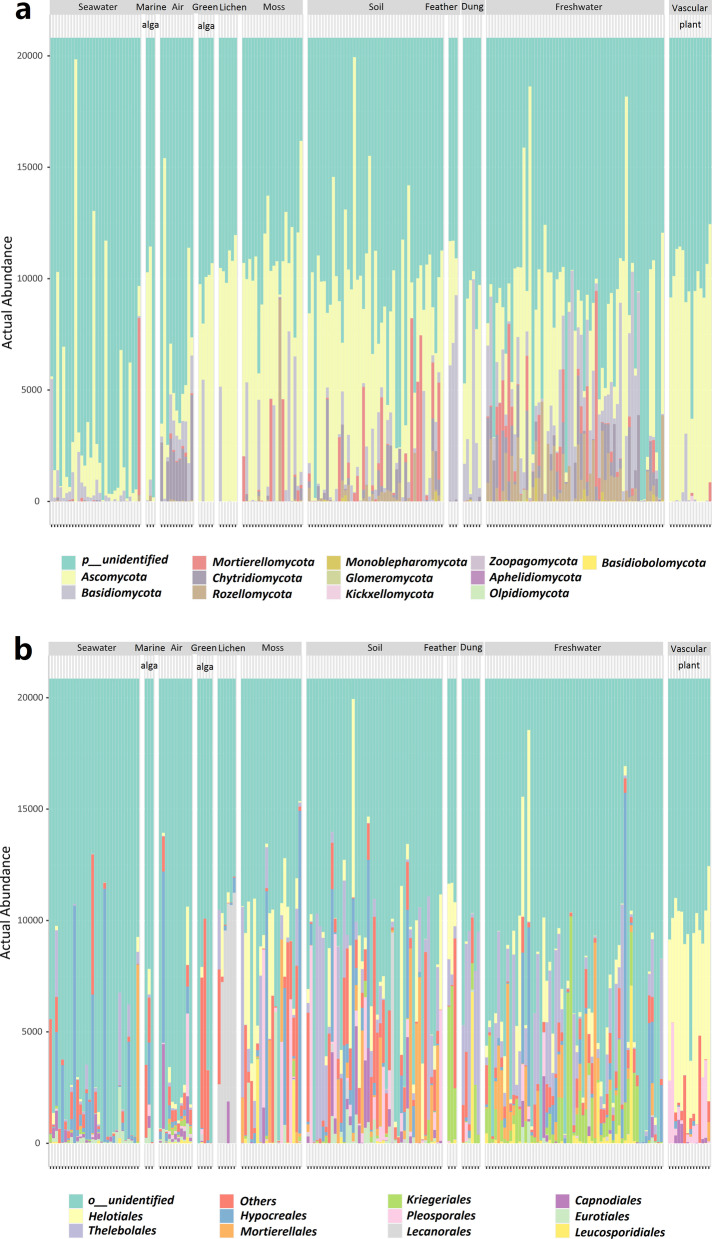
Bar plots showing the abundance of (**a**) fungal phyla, (**b**) fungal orders in the 202 samples collected from eleven habitats in the Fildes Region (maritime Antarctica)

The stacked bar plots showed that the fungal community compositions differed among the eleven habitats (i.e., air, soil, seawater, vascular plant, dung, moss, marine alga, lichen, green alga, freshwater, feather) (Fig. 2). For example, reads assigned to unknown phylum accounted for the largest proportion of reads in samples, especially in seawater and air. There were more phyla in freshwater than in other habitats (Fig. 2a). At the order level, *Helotiales* accounted for the largest proportion among the orders in vascular plant, while *Thelebolales* predominated in dung, *Kriegeriales* in feather, and *Lecanorales* in lichen (Fig. 2b).

### Differentiation between fungal assemblages across habitats

**Fig. 3:**
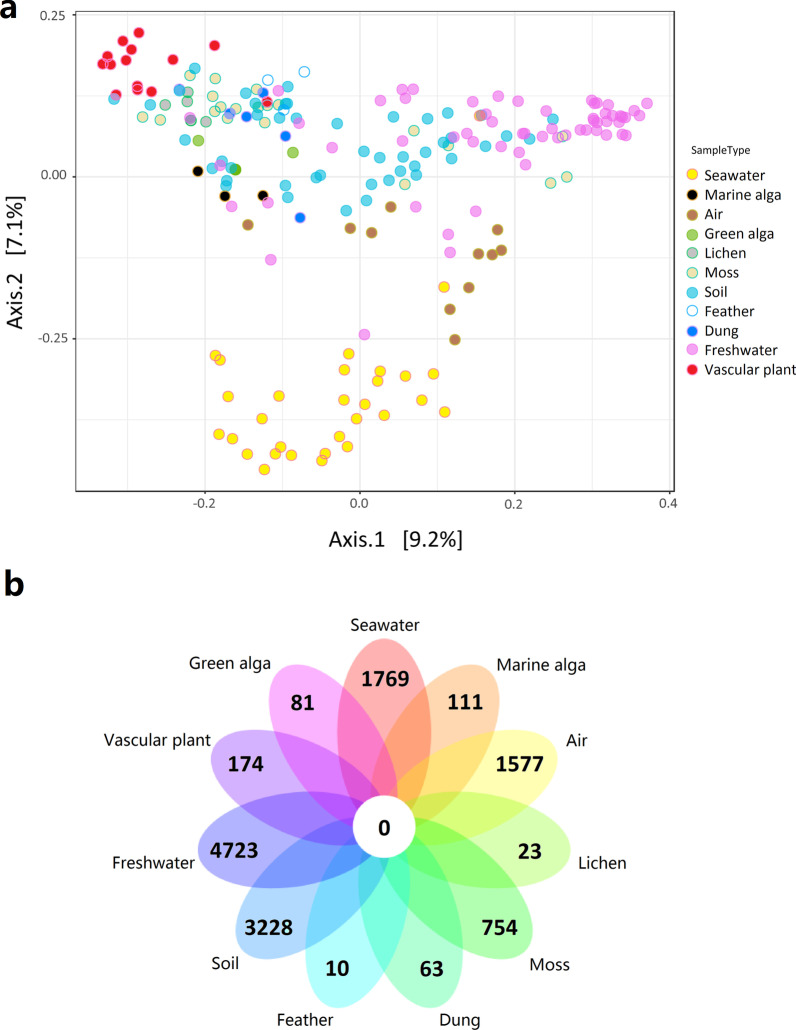
**a** Principal Coordinates Analysis (PCoA) ordination plot showing spatial pattern of fungal communities in the 202 samples from the eleven habitats; **b** Venn diagram showing the number of ASVs in the eleven habitats.

The PCoA diagram showed that the spatial patterns of the fungal communities were highly related to habitat types (Fig. 3a). ANOSIM tests also indicated the fungal community compositions were significantly different among the eleven different habitats (*R* = 0.5153, *p* < 0.001). Additionally, ANOSIM tests for the pairwise comparisons revealed the different degrees of similarity of fungal communities among different habitats (Table 1). For example, soil and air did not harbor significantly different fungal communities (*R* = 0.064393, *p* > 0.05), whereas fungal communities in air were significantly different from those in green alga (*R* = 0.91608, *p* < 0.001). A Venn diagram indicated none of ASVs were shared by all eleven habitats (Fig. 3b). In addition, Dendrogram analysis revealed the relationships of fungal communities among 202 samples collected from the eleven habitats, indicating that the samples clustered by habitat types (Additional file 1: Fig. S1). For example, with regard to fungal community composition, seawater samples clustered together and were separated from the samples in other habitats.

**Table 1:** ANOSIM results for the pairwise comparisons of the similarity among different habitats in the Fildes Region (maritime Antarctica)

Habitat type	Soil	Freshwater	Seawater	Air	Feather	Dung	Marine alga	Moss	Lichen	Vascular plant	Green alga
Soil	–	0.4014***	0.54378***	0.064393	0.1582	0.072084	0.30631*	0.073806	0.49243***	0.12049*	0.57507**
Freshwater	0.4014***	–	0.66236***	0.28663**	0.34803*	0.55482***	0.8175***	0.46873***	0.86957***	0.74104***	0.83646***
Seawater	0.54378***	0.66236***	–	0.66664***	0.938***	0.91133***	0.91339***	0.83644***	0.986***	0.97284***	0.95646***
Air	0.064393	0.28663**	0.66664***	–	0.88715**	0.87056***	0.86416**	0.46456***	0.99957***	0.97803***	0.91608***
Feather	0.1582	0.34803*	0.938***	0.88715**	–	0.19136	0.96296	−0.10604	0.77778*	0.90983***	0.81538*
Dung	0.072084	0.55482***	0.91133***	0.87056***	0.19136	–	0.79012*	0.17528	0.75926**	0.92543***	0.71733***
Marine alga	0.30631*	0.8175***	0.91339***	0.86416**	0.96296	0.79012*	–	0.60207***	0.85185*	0.97112***	0.26154
Moss	0.073806	0.46873***	0.83644***	0.46456***	-0.10604	0.17528	0.60207***	–	0.40659**	0.48658***	0.6898***
Lichen	0.49243***	0.86957***	0.986***	0.99957***	0.77778*	0.75926**	0.85185*	0.40659**	–	0.98428***	0.576**
Vascular plant	0.12049*	0.74104***	0.97284***	0.97803***	0.90983***	0.92543***	0.97112***	0.48658***	0.98428***	–	0.93409***
Green alga	0.57507**	0.83646***	0.95646***	0.91608***	0.81538*	0.71733***	0.26154	0.6898***	0.576**	0.93409***	–

^*^0.01 < *p* ≤ 0.05, **0.001 < *p* ≤ 0.01, ***p ≤ 0.001

**Fig. 4:**
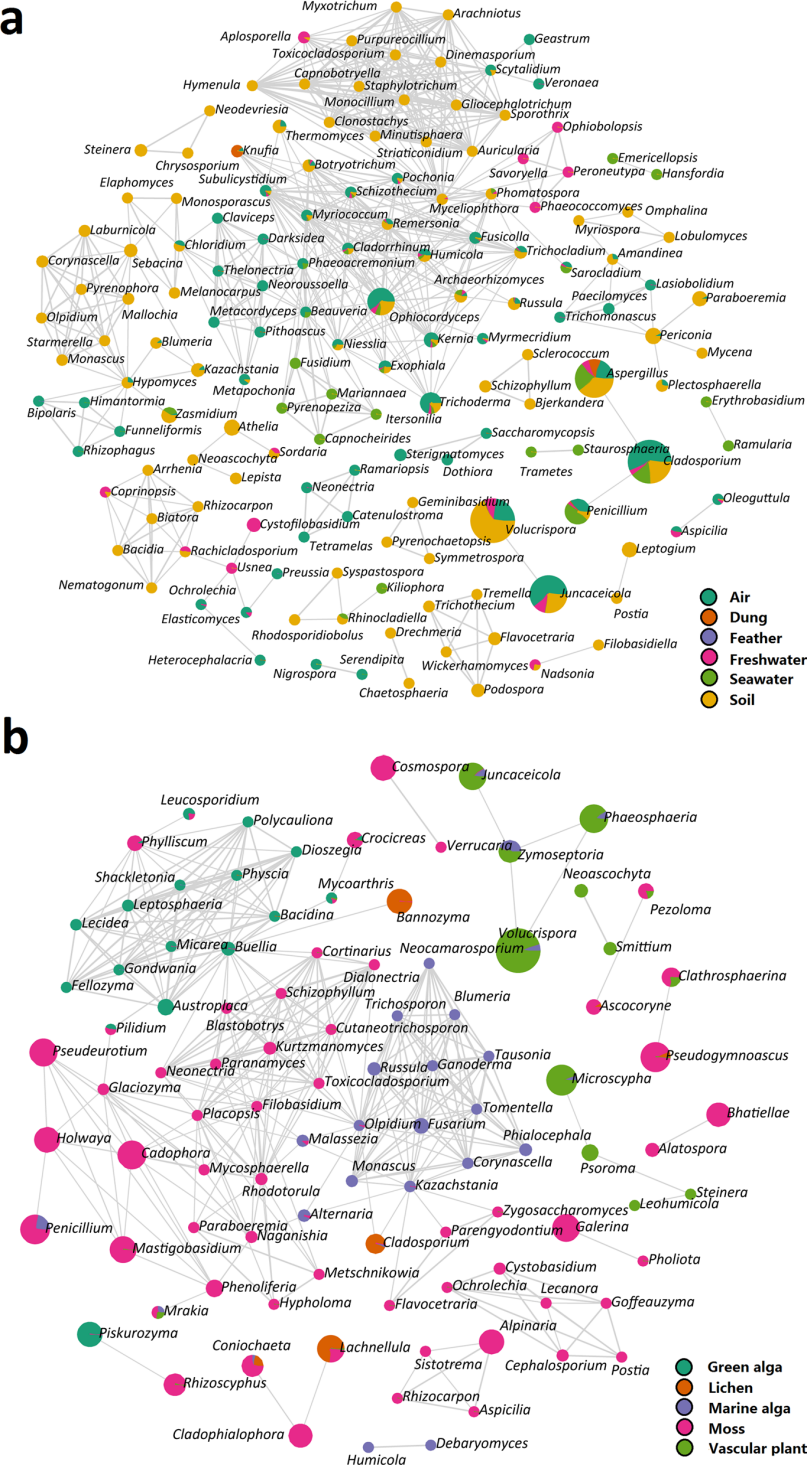
Correlation network analyses showing the correlation of fungal genera among (**a)** six habitat types (i.e., air, dung, feather, freshwater, seawater, and soil), (**b)** five habitat types (i.e., green alga, lichen, marine alga, moss, and vascular plant). Each node represents a fungal genus and the size of a node is based on sequence number. The nodes are colored based on habitat types

The co-occurrence patterns showed that the correlations between the fungal genera were highly related to habitats (Fig. 4). For example, five fungal genera (i.e., *Tremella, Trichothecium, Wickerhamomyces, Podospora,* and *Flavocetraria*) were in one module with more connections and occurred in the soil habitat (Fig. 4a), whereas two fungal genera (i.e., *Humicola* and *Debaryomyces*) were in another module and occurred in the marine alga habitat (Fig. 4b).

**Fig. 5:**
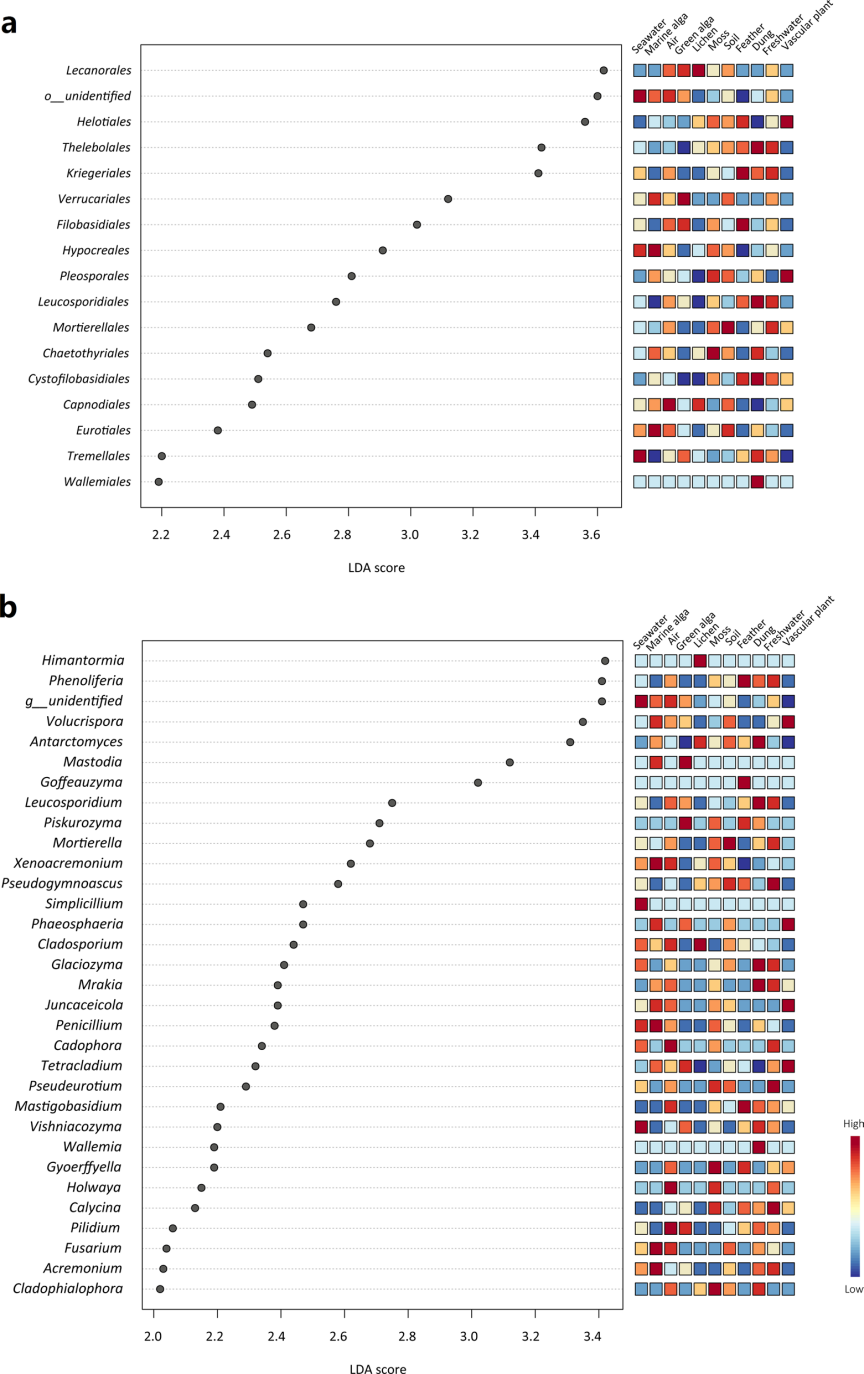
LEfSe analysis showing (**a**) the fungal orders and (**b**) genera that differ significantly among the eleven habitat types in the Fildes Region. Significant orders or genera are ranked by their LDA scores (x-axis). The right heatmap shows whether the relative abundances of orders or genera are higher (red) or lower (blue)

Many taxonomic groups showed habitat specificity in LEfse analysis (Fig. 5, Additional file 1: Figs. S2, S3, and S4). For example, phylum *Ascomycota* dominated in the vascular plant and lichen habitats, phylum *Basidiomycota* in the feather and dung habitats, and phylum *Chytridiomycota* in the air and freshwater habitats (Additional file 1: Fig. S2). Order *Lecanorales* was dominated in the lichen and green alga habitats, and order *Helotiales* in the vascular plant habitat. Moreover, order *Wallemiales* dominated in the dung habitat, and order *Kriegeriales* in the feather habitat (Fig. 5a). Genus *Phenoliferia* and *Goffeauzyma* dominated in the feather habitat, and genus *Simplicillium* in the seawater habitat; genera *Mastodia* and *Piskurozyma* were more abundant in the green alga habitat; genus *Himantormia* was more abundant in the lichen habitat; genus *Antarctomyces* was more abundant in the dung habitat (Fig. 5b).

### Ecological traits of fungal communities in various habitats

In terms of fungal ecological trait, 20 functional guilds and 6 growth forms were detected in this study. Using Fungaltratis, 2481 fungal ASVs identified at generic level were given guild assignments. A majority were assigned as saprophytic fungi (1899 ASVs), followed by plant pathogens (231 ASVs) and lichenized fungi (138 ASVs) (Additional file 4: Table S4).

**Fig. 6:**
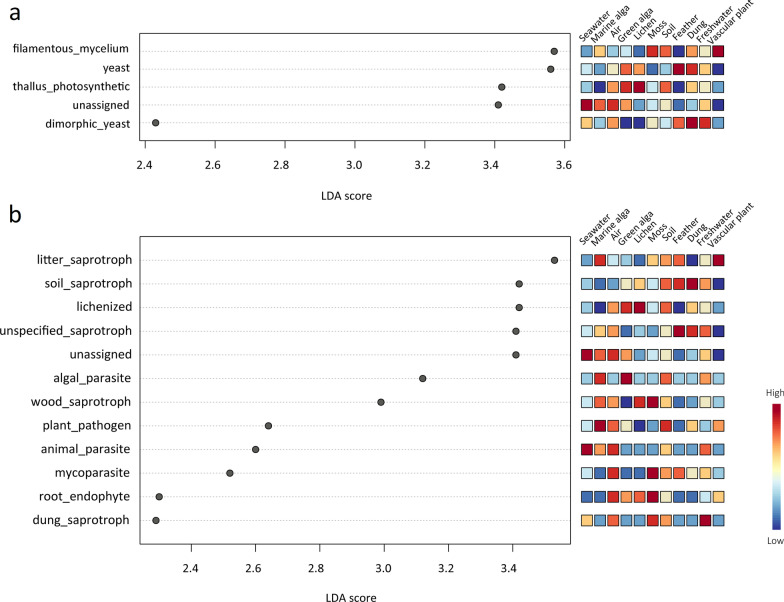
LEfSe analysis showing (**a**) the growth forms and (**b**) functional guilds that differ significantly among the eleven habitat types in the Fildes Region. Significant growth forms or lifestyles are ranked by their LDA scores (x-axis). The right heatmap shows whether the relative abundances of growth forms or functional guilds are higher (red) or lower (blue)

By using LEfse analysis, many ecological traits were significantly distinguished among different habitats. With regards to growth form, yeast predominated in the feather and dung habitats, but had low contributions in the moss and vascular plant habitats; thallus photosynthetic predominated in the lichen and green alga habitats (Fig. 6a). With regard to functional guild, lichenized fungi dominated in the lichen habitat, while had low contributions in the marine alga and feather habitats; algal parasite fungi dominated in the green alga and marine alga habitats (Fig. 6b).

A total of 43 fungal species were detected as potential human pathogens (Additional file 1: Table S5). The common species were *Pseudogymnoascus destructans* (68 samples)*, Malassezia restricta* (61 samples), *Alternaria tenuissima* (33 samples), *Cladosporium cladosporioides* (30 samples), *Parengyodontium album* (21 samples), *Aspergillus sydowii* (18 samples), *Aspergillus penicillioides* (16 samples), and *Fusarium solani* (11 samples)*.* In contrast, several fungal species (e.g., *Acremonium spinosum*, *Wickerhamomyces anomalus*) were infrequently detected.

## Discussion

Antarctic ice-free areas are a unique laboratory for understanding cold adaptation, the spread, and the colonization of microbes in extreme habitats. To date, the majority of studies on fungal communities have been conducted based on single habitat in the Antarctic ice-free areas and a comprehensive study involving various habitats remains scarce.

In this study, a total of 14,814 fungal ASVs, 313 known genera, and 320 known species were detected from the eleven habitats in the Fildes Region (maritime Antarctica). In previous studies that used next-generation sequencing, 87 genera and 123 species were detectable in the Antarctic soils [10]; Rosa et al. [17] identified 186 fungal amplicon sequence variants (ASVs) from air collected from King George Island (marine Antarctica); Rosa et al. [11] identified 346 fungal ASVs from soil samples in Deception Island (marine Antarctica). A total of 12 known phyla, including *Ascomycota*, *Basidiomycota*, *Chytridiomycota*, *Mortierellomycota*, *Rozellomycota*, *Monoblepharomycota*, *Glomeromycota*, *Kickxellomycota*, *Zoopagomycota*, *Aphelidiomycota*, *Olpidiomycota*, and *Basidiobolomycota*, were detected in this study. In a previous study, the phyla *Ascomycota*, *Mortierellomycota*, *Basidiomycota*, *Chytridiomycota*, *Rozellomycota*, *Mucoromycota*, *Calcarisporiellomycota*, and *Zoopagomycota* were detected in the soils from maritime Antarctica using DNA metabarcoding [13]. The phylum *Ascomycota* was dominant in Antarctic glacial ice fragments, followed by *Basidiomycota* and *Mortierellomycota* as revealed by amplicon-metagenome analysis [40]. de Souza et al. [18] found phyla *Ascomycota*, *Basidiomycota*, *Mortierellomycota*, *Chytridiomycota*, and *Rozellomycota* in two Antarctic lakes using amplicon-metagenome analysis. Overall, our data provided a considerably more comprehensive exploration of the fungal diversity in the Antarctic ice-free area.

Antarctica has a number of important environmental pressures (i.e., temperature, solar radiation, salinity, soil parameters, pH), which may be an important factor affecting fungal community composition. In this study, we found that fungal communities were significantly different among the eleven habitats. At a local scale, high habitat specificity in fungal communities may be attributable to the differences in current habitat factors (e.g., physicochemical factors, nutrient contents, antagonistic factors) or historical factors (e.g., availability for fungal colonization). For example, vegetation micro-niche availability (e.g., green alga in the moist niches and lichens in the dry niches) and physiological attributes (i.e., chemical defenses, or nutrient contents) may directly affect fungal communities in different hosts (i.e., lichen, moss, vascular plant, green alga, and marine alga) [41, 42]. Solar radiation is an important environmental factor affecting the composition of Antarctic fungal communities [43] and different habitats in the Fildes Region (e.g., soil, air, lichen thallus, or plant tissue), have different levels of solar radiation. The environmental conditions of marine habitats (e.g., salinity) are very different from those of terrestrial habitats and thereby fungal communities in freshwater and seawater were significantly different.

In this study, many fungal guilds were significantly different among various habitats. The most prevalent fungal guild identified in this study was saprotroph (e.g., soil saprotroph, litter saprotroph, wood saprotroph) (Additional file 4: Table S4). In soils, saprotroph fungi play an important ecological role in organic matter decomposition and nutrient cycling through their secretion of extracellular enzyme activities [44]. In seawater, fungi as saprotrophs, interact with marine phytoplankton and can have a significant impact on primary production dynamics and carbon flux in the marine food chain [45]. We found lichenized (functional guild), thallus photosynthetic (growth form), and *Lecanorales* (fungal order consisting mainly of lichenized taxa) dominated in the lichen habitat (Figs. 5a and 6). In this ice-free area of maritime Antarctica, lichenized fungi, as dominant vegetation, are the main supports for primary production (capable of supporting photosynthesis) [46]. In addition, *Kriegeriales* (order consisting mainly of yeast taxa) and yeast (growth form) predominated in the feather habitat (Figs. 5a and 6a). In our unpublished data, the colonization rate of cultured yeasts was also higher in feather samples than in other sample types.

Temperature changes caused by global climate change are an important factor affecting the composition of fungal communities. Previous studies have shown that warming leads to significant changes in fungal abundance [5, 6]. In the soils of maritime Antarctica, the richness, relative abundance, and composition of fungal guilds and growth forms are influenced by air temperature and edaphic factors [21]. In addition, new ice-free areas and new climatically suitable habitats may facilitate the establishment of fungal species, either naturally (e.g., lichen-, plant-, moss-, green alga-, air-, and seawater-associated fungi) or by the accidental introduction from animals (e.g., penguin-, bird-, and human-associated fungi). It is estimated that climate warming in marine Antarctica may lead to changes of habitats in ice-free areas, and then affects whole fungal communities in the ice-free area.

Climate warming in the Antarctic regions may also increase the risk of fungal diseases. Schütte et al. [47] noted an increase in the relative abundance of potential fungal pathogens after the thawing of the permafrost in Alaska. According to the Atlas of Clinical Fungi [39], this study revealed the broad spectrum of potential fungal pathogens of humans (43 species) in an Antarctic ice-free area. Previous studies have revealed the occurrence of potential fungal pathogens in the Antarctic environments, including *Aspergillus fumigatus*, *Byssochlamys spectabilis*, *Chrysosporium keratinophilum*, *Cryptococcus laurentii*, *Penicillium chrysogenum*, *Rhizopus oryzae*, and *Rhodotorula mucilaginosa*, which were isolated from ornithogenic soils and displayed virulence capabilities [48]. Our results based on next-generation sequencing provide an indicator of the potential health risk and further analyses of fungal isolates are needed to assess their virulences which are crucial for pathogenicity.

## Conclusion

The present study reveals the high diversity of fungal communities in the eleven different habitats and elucidates the ecological traits of fungal communities in an Antarctica ice-free area. We thereby conclude that habitat specificity rather than habitat overlap determined the distribution of fungal communities, suggesting that although fungal communities were connected by dispersal at the local scale, the environmental filter is a key factor driving fungal assemblages in this Antarctic ice-free area.

## Supplementary Information

**Additional file 1.** **Table S1.** Information on the 213 samples collected from the Fildes Region (maritime Antarctica). **Table S5.** An overview of potentially pathogenic fungi found in the eleven habitats from the Fildes Region (maritime Antarctica). **Fig. S1.** Dendrogram showing fungal communities in the 202 samples of eleven habitats from the Fildes Region (maritime Antarctica). **Fig. S2.** LEfSe analysis showing the fungal phyla that are significantly different among the eleven habitats in the Fildes Region (maritime Antarctica). Significant phyla are ranked by their LDA scores (x-axis). The right heatmap shows whether the relative abundances of phyla are higher (red) or lower (blue). **Fig. S3.** LEfSe analysis showing the fungal classes that are significantly different among the eleven habitats in the Fildes Region (maritime Antarctica). Significant classes are ranked by their LDA scores (x-axis). The right heatmap shows whether the relative abundances of classes are higher (red) or lower (blue). **Fig. S4.** LEfSe analysis showing the fungal families that are significantly different among the eleven habitats in the Fildes Region (maritime Antarctica). Significant families are ranked by their LDA scores (x-axis). The right heatmap shows whether the relative abundances of families are higher (red) or lower (blue).

**Additional file 2.** **Table S2.** Representative sequence of fungal ASV detected in the 213 samples.

**Additional file 3.** **Table S3.** Information on the 17,236 fungal ASVs in the 213 samples collected from the Fildes Region (maritime Antarctica).

**Additional file 4.** **Table S4.** Information on the 14,814 fungal ASVs in the 202 samples collected from the Fildes Region (maritime Antarctica).

## Data Availability

The datasets generated during and/or analysed during the current study are available in the National Center for Biotechnology Information under the BioProject ID PRJNA509411.
