# Supplementary material for: A comprehensive assessment of fungal communities in various habitats from an ice-free area of maritime Antarctica: diversity, distribution, and ecological trait
Source: Environ Microbiome. 2022 Nov 15;17:54. doi: 10.1186/s40793-022-00450-0 (PMC9667611; doi:10.1186/s40793-022-00450-0)
Supplement: Supplementary file 1 — Additional file 1. Table S1. Information on the 213 samples collected from the Fildes Region (maritime Antarctica). Table S5. An overview of potentially pathogenic fungi found in the eleven habitats from the Fildes Region (maritime Antarctica). Fig. S1. Dendrogram showing fungal communities in the 202 samples of eleven habitats from the Fildes Region (maritime Antarctica). Fig. S2. LEfSe analysis showing the fungal phyla that are significantly different among the eleven habitats in the Fildes Region (maritime Antarctica). Significant phyla are ranked by their LDA scores (x-axis). The right heatmap shows whether the relative abundances of phyla are higher (red) or lower (blue). Fig. S3. LEfSe analysis showing the fungal classes that are significantly different among the eleven habitats in the Fildes Region (maritime Antarctica). Significant classes are ranked by their LDA scores (x-axis). The right heatmap shows whether the relative abundances of classes are higher (red) or lower (blue). Fig. S4. LEfSe analysis showing the fungal families that are significantly different among the eleven habitats in the Fildes Region (maritime Antarctica). Significant families are ranked by their LDA scores (x-axis). The right heatmap shows whether the relative abundances of families are higher (red) or lower (blue). [file 40793_2022_450_MOESM1_ESM.docx]

**Table S1.** Information on the 213 samples collected from the Fildes Region (maritime Antarctica).

| no. | Sampling code | Habitat type | Raw reads | Trimmed reads | Subsampled reads | Number of fungal ASVs | NCBI SRA Number |
| --- | --- | --- | --- | --- | --- | --- | --- |
| 1 | A1_0 | seawater (0 m in Ardley Cove) | 36963 | 32346 | 20812 | 83 | In this study |
| 2 | A1_10 | seawater (-10 m in Ardley Cove) | 32660 | 28334 | 20812 | 256 | In this study |
| 3 | A1_30 | seawater (-30 m in Ardley Cove) | 32712 | 27142 | 20812 | 330 | In this study |
| 4 | A2_0 | seawater (0 m in Ardley Cove) | 42910 | 30523 | 20812 | 433 | In this study |
| 5 | A3_0 | seawater (0 m in Ardley Cove) | 31878 | 28060 | 20812 | 64 | In this study |
| 6 | A3_10 | seawater (-10 m in Ardley Cove) | 33570 | 27246 | 20812 | 68 | In this study |
| 7 | A3_5 | seawater (-5 m in Ardley Cove) | 46283 | 41958 | 20812 | 112 | In this study |
| 8 | A4_0 | seawater (0 m in Ardley Cove) | 35221 | 31095 | 20812 | 149 | In this study |
| 9 | A4_10 | seawater (-10 m in Ardley Cove) | 43598 | 39784 | 20812 | 206 | In this study |
| 10 | A4_30 | seawater (-30 m in Ardley Cove) | 44684 | 40934 | 20812 | 36 | In this study |
| 11 | A4_5 | seawater (-5 m in Ardley Cove) | 35775 | 32358 | 20812 | 165 | In this study |
| 12 | A5_0 | seawater (0 m in Ardley Cove) | 39165 | 34479 | 20812 | 214 | In this study |
| 13 | A5_10 | seawater (-10 m in Ardley Cove) | 33454 | 23972 | 20812 | 218 | In this study |
| 14 | A5_5 | seawater (-5 m in Ardley Cove) | 44145 | 38429 | 20812 | 112 | In this study |
| 15 | G1_0 | seawater (0 m in Great Wall Cove) | 37464 | 32587 | 20812 | 110 | In this study |
| 16 | G1_10 | seawater (-10 m in Great Wall Cove) | 48569 | 43659 | 20812 | 186 | In this study |
| 17 | G1_20 | seawater (-20 m in Great Wall Cove) | 43068 | 38400 | 20812 | 172 | In this study |
| 18 | G1_5 | seawater (-5 m in Great Wall Cove) | 53850 | 43922 | 20812 | 43 | In this study |
| 19 | G2_0 | seawater (0 m in Great Wall Cove) | 39485 | 21546 | 20812 | 49 | In this study |
| 20 | G3_0 | seawater (0 m in Great Wall Cove) | 37183 | 30606 | 20812 | 156 | In this study |
| 21 | G3_5 | seawater (-5 m in Great Wall Cove) | 57983 | 52985 | 20812 | 59 | In this study |
| 22 | G4_0 | seawater (0 m in Great Wall Cove) | 41082 | 29145 | 20812 | 83 | In this study |
| 23 | G4_10 | seawater (-10 in Great Wall Cove) | 40099 | 32702 | 20812 | 50 | In this study |
| 24 | G4_5 | seawater (-5 m in Great Wall Cove) | 57157 | 51376 | 20812 | 87 | In this study |
| 25 | G5_0 | seawater (0 m in Great Wall Cove) | 52094 | 46175 | 20812 | 157 | In this study |
| 26 | G5_10 | seawater (-10 m in Great Wall Cove) | 39670 | 33707 | 20812 | 99 | In this study |
| 27 | G5_20 | seawater (-20 m in Great Wall Cove) | 32924 | 29291 | 20812 | 74 | In this study |
| 28 | G5_30 | seawater (-30 m in Great Wall Cove) | 38916 | 21642 | 20812 | 38 | In this study |
| 29 | G5_5 | seawater (-5 m in Great Wall Cove) | 44295 | 40532 | 20812 | 41 | In this study |
| 30 | NW43 | Seawater (0 m near shore in Fildes Strait) | 104056 | 99806 | 20812 | 124 | In this study |
| 31 | NA01 | Marine alga (*Pachymenia orbicularis*) | 44822 | 83 | - | - | In this study |
| 32 | NA02 | Marine alga (*Adenocystis utricularis*) | 57931 | 44336 | 20812 | 34 | In this study |
| 33 | NA03 | Marine alga (*Palmaria decipiens*) | 37599 | 7 | - | - | In this study |
| 34 | NA04 | Marine alga (*Pachymenia orbicularis*) | 34043 | 28359 | 20812 | 138 | In this study |
| 35 | NA05 | Marine alga (*Pachymenia orbicularis*) | 43950 | 40408 | 20812 | 8 | In this study |
| 36 | NK01 | Air | 52696 | 43901 | 20812 | 115 | In this study |
| 37 | NK02 | Air | 38241 | 20830 | 20812 | 281 | In this study |
| 38 | NK03 | Air | 42406 | 37804 | 20812 | 106 | In this study |
| 39 | NK04 | Air | 48079 | 42232 | 20812 | 369 | In this study |
| 40 | NK05 | Air | 40709 | 35851 | 20812 | 291 | In this study |
| 41 | NK06 | Air | 43128 | 38259 | 20812 | 334 | In this study |
| 42 | NK07 | Air | 53225 | 47986 | 20812 | 249 | In this study |
| 43 | NK08 | Air | 47819 | 41239 | 20812 | 291 | In this study |
| 44 | NK09 | Air | 54711 | 47998 | 20812 | 408 | In this study |
| 45 | NK10 | Air | 38671 | 32519 | 20812 | 110 | In this study |
| 46 | NK11 | Air | 50584 | 44251 | 20812 | 278 | In this study |
| 47 | NB01 | Green alga (*Prasiola crispa*) | 30467 | 25410 | 20812 | 7 | In this study |
| 48 | NB02 | Green alga (*Prasiola crispa*) | 32391 | 27855 | 20812 | 114 | In this study |
| 49 | NB03 | Green alga (*Prasiola crispa*) | 56731 | 43780 | 20812 | 2 | In this study |
| 50 | NB04 | Green alga (*Prasiola crispa*) | 48208 | 32016 | 20812 | 20 | In this study |
| 51 | NB05 | Green alga (*Prasiola crispa*) | 46693 | 35330 | 20812 | 6 | In this study |
| 52 | NL01 | Lichen (*Sphaerophorus globosus*) | 54082 | 43170 | 20812 | 14 | In this study |
| 53 | NL02 | Lichen (*Sphaerophorus globosus*) | 51486 | 38304 | 20812 | 18 | In this study |
| 54 | NL03 | Lichen (*Himantormia lugubris*) | 51069 | 28907 | 20812 | 7 | In this study |
| 55 | NL06 | Lichen (*Sphaerophorus globosus*) | 42006 | 29215 | 20812 | 11 | In this study |
| 56 | NL07 | Lichen (*Stereocaulon alpinum*) | 39113 | 19269 | - | - | In this study |
| 57 | NL08 | Lichen (*Stereocaulon alpinum*) | 31025 | 15562 | - | - | In this study |
| 58 | NL09 | Lichen (*Himantormia lugubris*) | 64580 | 42854 | 20812 | 6 | In this study |
| 59 | NL10 | Lichen (*Stereocaulon alpinum*) | 35402 | 17251 | - | - | In this study |
| 60 | NL11 | Lichen (*Himantormia lugubris*) | 61515 | 41605 | 20812 | 6 | In this study |
| 61 | NM01 | Moss (*Andreaea rupestris*) | 33829 | 31865 | 20812 | 41 | PRJNA448984 |
| 62 | NM02 | Moss (*Polytrichastrum alpinum*) | 35739 | 31932 | 20812 | 179 | PRJNA448984 |
| 63 | NM03 | Moss (*Polytrichastrum alpinum*) | 39619 | 34646 | 20812 | 55 | PRJNA448984 |
| 64 | NM04 | Moss (*Andreaea rupestris*) | 36875 | 26768 | 20812 | 56 | PRJNA448984 |
| 65 | NM05 | Moss (*Polytrichastrum alpinum*) | 36712 | 32303 | 20812 | 40 | PRJNA448984 |
| 66 | NM06 | Moss (*Sanionia uncinata*) | 33831 | 23450 | 20812 | 93 | PRJNA448984 |
| 67 | NM07 | Moss (*Sanionia uncinata*) | 41314 | 36387 | 20812 | 38 | PRJNA448984 |
| 68 | NM08 | Moss (*Andreaea rupestris*) | 33498 | 29042 | 20812 | 41 | PRJNA448984 |
| 69 | NM09 | Moss (*[Warnstorfia exannulata](https://blast.ncbi.nlm.nih.gov/Blast.cgi" \l "alnHdr_63191686" \o "Go to alignment for Warnstorfia exannulata isolate MDP439 small ribosomal protein subunit 4 (rps4) gene, partial cds; and tRNA-Ser gene, partial sequence; chloroplast)*) | 36570 | 27825 | 20812 | 152 | PRJNA448984 |
| 70 | NM10 | Moss (*Polytrichastrum alpinum*) | 30518 | 28524 | 20812 | 26 | PRJNA448984 |
| 71 | NM11 | Moss (*Platyneuron laticostatum*) | 32749 | 29810 | 20812 | 63 | PRJNA448984 |
| 72 | NM12 | Moss (*Andreaea rupestris*) | 31147 | 25293 | 20812 | 14 | PRJNA448984 |
| 73 | NM13 | Moss (*Sanionia uncinata*) | 43280 | 38294 | 20812 | 100 | PRJNA448984 |
| 74 | NM14 | Moss (*Syntrichia* sp.) | 31673 | 29328 | 20812 | 24 | PRJNA448984 |
| 75 | NM15 | Moss (*Andreaea rupestris*) | 30400 | 26982 | 20812 | 14 | PRJNA448984 |
| 76 | NM16 | Moss (*Sanionia uncinata*) | 30470 | 26992 | 20812 | 58 | PRJNA448984 |
| 77 | NM17 | Moss (*Andreaea rupestris*) | 37534 | 33750 | 20812 | 40 | PRJNA448984 |
| 78 | NM18 | Moss (*[Warnstorfia exannulata](https://blast.ncbi.nlm.nih.gov/Blast.cgi" \l "alnHdr_63191686" \o "Go to alignment for Warnstorfia exannulata isolate MDP439 small ribosomal protein subunit 4 (rps4) gene, partial cds; and tRNA-Ser gene, partial sequence; chloroplast)*) | 41842 | 35685 | 20812 | 342 | PRJNA448984 |
| 79 | NM19 | Moss (*Polytrichastrum alpinum*) | 38094 | 33614 | 20812 | 63 | PRJNA448984 |
| 80 | NM20 | Moss (*[Warnstorfia exannulata](https://blast.ncbi.nlm.nih.gov/Blast.cgi" \l "alnHdr_63191686" \o "Go to alignment for Warnstorfia exannulata isolate MDP439 small ribosomal protein subunit 4 (rps4) gene, partial cds; and tRNA-Ser gene, partial sequence; chloroplast)*) | 40153 | 32864 | 20812 | 200 | PRJNA448984 |
| 81 | AN01 | Soil (without vegetation) | 57522 | 30149 | 20812 | 337 | PRJNA445173 |
| 82 | AN02 | Soil (without vegetation) | 53191 | 47782 | 20812 | 37 | PRJNA445173 |
| 83 | AN03 | Soil (without vegetation) | 43651 | 32933 | 20812 | 70 | PRJNA445173 |
| 84 | AN04 | Soil (without vegetation) | 51379 | 45482 | 20812 | 57 | PRJNA445173 |
| 85 | AN05 | Soil (without vegetation) | 50374 | 42876 | 20812 | 31 | PRJNA445173 |
| 86 | AN06 | Soil (without vegetation) | 73617 | 62502 | 20812 | 204 | PRJNA445173 |
| 87 | AN07 | Soil (without vegetation) | 47616 | 39507 | 20812 | 217 | PRJNA445173 |
| 88 | AN08 | Soil (without vegetation) | 55531 | 45509 | 20812 | 133 | PRJNA445173 |
| 89 | AN09 | Soil (without vegetation) | 60657 | 49765 | 20812 | 67 | PRJNA445173 |
| 90 | AN10 | Soil (without vegetation) | 55745 | 34738 | 20812 | 44 | PRJNA445173 |
| 91 | AN11 | Soil (without vegetation) | 46762 | 16373 | - | - | PRJNA445173 |
| 92 | AN12 | Soil (without vegetation) | 40794 | 35567 | 20812 | 69 | PRJNA445173 |
| 93 | AN13 | Soil (without vegetation) | 58608 | 52597 | 20812 | 138 | PRJNA445173 |
| 94 | AN14 | Soil (without vegetation) | 59292 | 49640 | 20812 | 93 | PRJNA445173 |
| 95 | NS01 | Soil (without vegetation) | 46705 | 41192 | 20812 | 82 | PRJNA448984 |
| 96 | NS07 | Soil (without vegetation) | 42000 | 32962 | 20812 | 35 | PRJNA448984 |
| 97 | NS09 | Soil (without vegetation) | 31079 | 26285 | 20812 | 47 | PRJNA448984 |
| 98 | NS14 | Soil (without vegetation) | 34878 | 23339 | 20812 | 309 | PRJNA448984 |
| 99 | NS17 | Soil (without vegetation) | 51054 | 47580 | 20812 | 48 | PRJNA448984 |
| 100 | NS22 | Soil (without vegetation) | 42162 | 33160 | 20812 | 281 | PRJNA448984 |
| 101 | NS28 | Soil (without vegetation) | 38758 | 26148 | 20812 | 110 | In this study |
| 102 | NS29 | Soil (without vegetation) | 35546 | 30370 | 20812 | 84 | PRJNA448984 |
| 103 | NS30 | Soil (without vegetation) | 42619 | 31401 | 20812 | 129 | PRJNA448984 |
| 104 | NS31 | Soil (without vegetation) | 31975 | 26165 | 20812 | 102 | In this study |
| 105 | NS38 | Soil (without vegetation) | 37849 | 30903 | 20812 | 247 | In this study |
| 106 | NS41 | Soil (without vegetation) | 53314 | 46413 | 20812 | 59 | PRJNA448984 |
| 107 | NS43 | Soil (without vegetation) | 48680 | 38860 | 20812 | 58 | PRJNA448984 |
| 108 | NS46 | Soil (without vegetation) | 42039 | 34286 | 20812 | 110 | PRJNA448984 |
| 109 | NS50 | Soil (without vegetation) | 31152 | 6364 | - | - | In this study |
| 110 | NS52 | Soil (without vegetation) | 42831 | 27499 | 20812 | 286 | PRJNA448984 |
| 111 | NS53 | Soil (without vegetation) | 38286 | 17928 | - | - | PRJNA448984 |
| 112 | NS55 | Soil (without vegetation) | 33324 | 19944 | - | - | PRJNA448984 |
| 113 | NS58 | Soil (without vegetation) | 34715 | 30267 | 20812 | 173 | PRJNA448984 |
| 114 | NS66 | Soil (without vegetation) | 38463 | 33595 | 20812 | 31 | PRJNA448984 |
| 115 | NS12 | Soil (without vegetation) | 39705 | 22740 | 20812 | 157 | In this study |
| 116 | NS13 | Soil (without vegetation) | 35391 | 31857 | 20812 | 65 | In this study |
| 117 | NS26 | Soil (without vegetation) | 35920 | 17128 | - | - | In this study |
| 118 | NS32 | Soil (without vegetation) | 40047 | 30315 | 20812 | 140 | In this study |
| 119 | NS35 | Soil (without vegetation) | 39057 | 23656 | 20812 | 168 | In this study |
| 120 | NS36 | Soil (without vegetation) | 35688 | 30835 | 20812 | 134 | In this study |
| 121 | NS47 | Soil (without vegetation) | 32226 | 29225 | 20812 | 5 | In this study |
| 122 | NS54 | Soil (without vegetation) | 33695 | 28236 | 20812 | 111 | In this study |
| 123 | NS57 | Soil (without vegetation) | 35172 | 10096 | - | - | In this study |
| 124 | NS05 | Soil (rhizosphere of *Deschampsia antarctica*) | 31098 | 25762 | 20812 | 48 | In this study |
| 125 | NS11 | Soil (rhizosphere of *Deschampsia antarctica*) | 38865 | 31984 | 20812 | 93 | In this study |
| 126 | NS16 | Soil (rhizosphere of *Deschampsia antarctica*) | 50268 | 44214 | 20812 | 16 | In this study |
| 127 | NS20 | Soil (rhizosphere of *Deschampsia antarctica*) | 37719 | 27050 | 20812 | 177 | In this study |
| 128 | NS25 | Soil (rhizosphere of *Deschampsia antarctica*) | 41517 | 24412 | 20812 | 135 | In this study |
| 129 | NS34 | Soil (rhizosphere of *Deschampsia antarctica*) | 41578 | 37572 | 20812 | 74 | In this study |
| 130 | NS37 | Soil (rhizosphere of *Deschampsia antarctica*) | 43364 | 41306 | 20812 | 43 | In this study |
| 131 | NS39 | Soil (rhizosphere of *Deschampsia antarctica*) | 39000 | 29675 | 20812 | 43 | In this study |
| 132 | NY01 | Feather | 60800 | 48800 | 20812 | 46 | In this study |
| 133 | NY02 | Feather | 64616 | 51726 | 20812 | 30 | In this study |
| 134 | NY03 | Feather | 45226 | 40203 | 20812 | 44 | In this study |
| 135 | NQ01 | Dung (Penguin) | 59581 | 48471 | 20812 | 35 | In this study |
| 136 | NQ02 | Dung (Penguin) | 56600 | 47402 | 20812 | 59 | In this study |
| 137 | NQ03 | Dung (Penguin) | 72723 | 64605 | 20812 | 52 | In this study |
| 138 | NQ04 | Dung (Penguin) | 61020 | 54640 | 20812 | 46 | In this study |
| 139 | NQ05 | Dung (Penguin) | 69115 | 26610 | 20812 | 10 | In this study |
| 140 | NQ06 | Dung (Penguin) | 61856 | 55048 | 20812 | 13 | In this study |
| 141 | NW01 | Freshwater (stream water) | 41141 | 37114 | 20812 | 277 | PRJNA448984 |
| 142 | NW02 | Freshwater (lake water) | 43942 | 39179 | 20812 | 174 | PRJNA448984 |
| 143 | NW04 | Freshwater (stream water) | 36998 | 27065 | 20812 | 240 | PRJNA448984 |
| 144 | NW05 | Freshwater (lake water) | 44773 | 40175 | 20812 | 75 | PRJNA448984 |
| 145 | NW06 | Freshwater (stream water) | 34111 | 28775 | 20812 | 445 | PRJNA448984 |
| 146 | NW07 | Freshwater (lake water) | 40304 | 26479 | 20812 | 236 | PRJNA448984 |
| 147 | NW08 | Freshwater (lake water) | 36654 | 31740 | 20812 | 221 | PRJNA448984 |
| 148 | NW09 | Freshwater (stream water) | 32223 | 28717 | 20812 | 255 | PRJNA448984 |
| 149 | NW11 | Freshwater (stream water) | 34527 | 30303 | 20812 | 222 | PRJNA448984 |
| 150 | NW14 | Freshwater (lake water) | 34944 | 29551 | 20812 | 50 | PRJNA448984 |
| 151 | NW15 | Freshwater (lake water) | 34711 | 30382 | 20812 | 202 | PRJNA448984 |
| 152 | NW17 | Freshwater (stream water) | 31704 | 22818 | 20812 | 181 | In this study |
| 153 | NW18 | Freshwater (stream water) | 39830 | 33490 | 20812 | 196 | PRJNA448984 |
| 154 | NW22 | Freshwater (lake water) | 36068 | 29134 | 20812 | 72 | PRJNA448984 |
| 155 | NW26 | Freshwater (stream water) | 40042 | 36588 | 20812 | 167 | PRJNA448984 |
| 156 | NW30 | Freshwater (lake water) | 50660 | 38421 | 20812 | 75 | PRJNA448984 |
| 157 | NW35 | Freshwater (stream water) | 48920 | 45207 | 20812 | 356 | PRJNA448984 |
| 158 | NW41 | Freshwater (lake water) | 131050 | 125173 | 20812 | 104 | PRJNA448984 |
| 159 | NW45 | Freshwater (stream water) | 111343 | 101943 | 20812 | 502 | PRJNA448984 |
| 160 | NW48 | Freshwater (lake water) | 92126 | 84890 | 20812 | 330 | PRJNA448984 |
| 161 | NW03 | Freshwater (snow melting water) | 36527 | 33952 | 20812 | 49 | In this study |
| 162 | NW10 | Freshwater (lake water) | 35125 | 31045 | 20812 | 190 | In this study |
| 163 | NW12 | Freshwater (lake water) | 30532 | 26203 | 20812 | 75 | In this study |
| 164 | NW16 | Freshwater (lake water) | 59060 | 53160 | 20812 | 112 | In this study |
| 165 | NW19 | Freshwater (lake water) | 35207 | 26829 | 20812 | 135 | In this study |
| 166 | NW20 | Freshwater (lake water) | 34992 | 32270 | 20812 | 40 | In this study |
| 167 | NW24 | Freshwater (stream water) | 33410 | 28681 | 20812 | 272 | In this study |
| 168 | NW25 | Freshwater (stream water) | 38639 | 32438 | 20812 | 179 | In this study |
| 169 | NW27 | Freshwater (lake water) | 44919 | 32898 | 20812 | 189 | In this study |
| 170 | NW28 | Freshwater (lake water) | 31113 | 26896 | 20812 | 155 | In this study |
| 171 | NW29 | Freshwater (lake water) | 33731 | 29046 | 20812 | 202 | In this study |
| 172 | NW32 | Freshwater (stream water) | 44619 | 28635 | 20812 | 65 | In this study |
| 173 | NW33 | Freshwater (snow melting water) | 93808 | 87215 | 20812 | 47 | In this study |
| 174 | NW34 | Freshwater (ice melting water) | 54553 | 47588 | 20812 | 165 | In this study |
| 175 | NW36 | Freshwater (lake water) | 66791 | 61859 | 20812 | 391 | In this study |
| 176 | NW37 | Freshwater (lake water) | 95934 | 77402 | 20812 | 187 | In this study |
| 177 | NW38 | Freshwater (lake water) | 46495 | 44202 | 20812 | 144 | In this study |
| 178 | NW39 | Freshwater (lake water) | 64420 | 54709 | 20812 | 138 | In this study |
| 179 | NW40 | Freshwater (lake water) | 95739 | 88623 | 20812 | 277 | In this study |
| 180 | NW42 | Freshwater (stream water) | 77988 | 73916 | 20812 | 88 | In this study |
| 181 | NW44 | Freshwater (lake water) | 38179 | 26009 | 20812 | 206 | In this study |
| 182 | NW46 | Freshwater (stream water) | 58560 | 52130 | 20812 | 476 | In this study |
| 183 | NW47 | Freshwater (stream water) | 65831 | 46089 | 20812 | 417 | In this study |
| 184 | NW49 | Freshwater (lake water) | 139071 | 122171 | 20812 | 178 | In this study |
| 185 | NW50 | Freshwater (lake water) | 50059 | 46745 | 20812 | 227 | In this study |
| 186 | NW51 | Freshwater (stream water) | 53525 | 47106 | 20812 | 88 | In this study |
| 187 | NW52 | Freshwater (stream water) | 42264 | 38148 | 20812 | 93 | In this study |
| 188 | NW53 | Freshwater (stream water) | 31363 | 26641 | 20812 | 420 | In this study |
| 189 | NW54 | Freshwater (stream water) | 56809 | 48007 | 20812 | 99 | In this study |
| 190 | NW55 | Freshwater (stream water) | 34265 | 24984 | 20812 | 214 | In this study |
| 191 | NW56 | Freshwater (snow melting water) | 46406 | 42250 | 20812 | 74 | In this study |
| 192 | NW57 | Freshwater (ice melting water) | 50061 | 44751 | 20812 | 28 | In this study |
| 193 | NW58 | Freshwater (ice melting water) | 36284 | 32466 | 20812 | 33 | In this study |
| 194 | NW59 | Freshwater (lake water) | 50947 | 46031 | 20812 | 86 | In this study |
| 195 | NW60 | Freshwater (lake water) | 48271 | 43025 | 20812 | 143 | In this study |
| 196 | NW61 | Freshwater (ice melting water) | 42314 | 23838 | 20812 | 394 | In this study |
| 197 | NW62 | Freshwater (lake water) | 50781 | 43895 | 20812 | 140 | In this study |
| 198 | NW63 | Freshwater (lake water) | 58499 | 53155 | 20812 | 75 | In this study |
| 199 | NW64 | Freshwater (lake water) | 58382 | 51988 | 20812 | 66 | In this study |
| 200 | P01 | Plant (aboveground parts of *Deschampsia antarctica*) | 44470 | 40852 | 20812 | 30 | PRJNA448984 |
| 201 | P04 | Plant (aboveground parts of *Deschampsia antarctica*) | 44982 | 40868 | 20812 | 50 | PRJNA448984 |
| 202 | P07 | Plant (aboveground parts of *Deschampsia antarctica*) | 46081 | 27756 | 20812 | 42 | PRJNA448984 |
| 203 | P10 | Plant (aboveground parts of *Deschampsia antarctica*) | 43000 | 38789 | 20812 | 31 | PRJNA448984 |
| 204 | P13 | Plant (aboveground parts of *Deschampsia antarctica*) | 56688 | 51497 | 20812 | 28 | PRJNA448984 |
| 205 | P16 | Plant (aboveground parts of *Deschampsia antarctica*) | 47535 | 43876 | 20812 | 24 | PRJNA448984 |
| 206 | P19 | Plant (aboveground parts of *Deschampsia antarctica*) | 35368 | 29300 | 20812 | 22 | PRJNA448984 |
| 207 | P22 | Plant (aboveground parts of *Deschampsia antarctica*) | 41590 | 38447 | 20812 | 32 | PRJNA448984 |
| 208 | P02 | Plant (roots of *Deschampsia antarctica*) | 47493 | 40959 | 20812 | 36 | In this study |
| 209 | P05 | Plant (roots of *Deschampsia antarctica*) | 45450 | 41488 | 20812 | 44 | In this study |
| 210 | P08 | Plant (roots of *Deschampsia antarctica*) | 59310 | 52791 | 20812 | 57 | In this study |
| 211 | P11 | Plant (roots of *Deschampsia antarctica*) | 52153 | 45135 | 20812 | 68 | In this study |
| 212 | P17 | Plant (roots of *Deschampsia antarctica*) | 50772 | 43900 | 20812 | 19 | In this study |
| 213 | P23 | Plant (roots of *Deschampsia antarctica*) | 44011 | 40715 | 20812 | 39 | In this study |

**Table S5.** An overview of potentially pathogenic fungi found in the eleven habitats from the Fildes Region (maritime Antarctica).

| Fungal species | Soil | Air | Seawater | Freshwater | Marine alga | Green alga | Vascular plant | Lichen | Moss | Dung | Feather | Total |
| --- | --- | --- | --- | --- | --- | --- | --- | --- | --- | --- | --- | --- |
| *Acremonium spinosum* | - | 1 | - | - | - | - | - | - | - | - | - | 1 |
| *Alternaria chlamydospora* | 1 | - | - | - | 1 | - | - | - | - | - | - | 2 |
| *Alternaria tenuissima* | 9 | 6 | 8 | 4 | - | - | - | - | 4 | 2 | - | 33 |
| *Aspergillus caesiellus* | - | 1 | - | - | - | - | - | - | - | - | - | 1 |
| *Aspergillus calidoustus* | 1 | - | - | - | - | - | - | - | - | - | - | 1 |
| *Aspergillus fumigatus* | 3 | 2 | - | - | - | - | - | - | - | - | - | 5 |
| *Aspergillus nidulans* | - | - | - | - | - | - | - | - | - | 1 | - | 1 |
| *Aspergillus penicillioides* | 6 | 1 | 3 | 1 | 1 | - | - | - | 1 | 3 | - | 16 |
| *Aspergillus restrictus* | - | - | - | 1 | - | - | - | - | - | 1 | - | 2 |
| *Aspergillus sydowii* | - | 5 | 13 | - | - | - | - | - | - | - | - | 18 |
| *Candida albicans* | - | - | - | 1 | - | - | - | - | - | - | - | 1 |
| *Candida blankii* | 2 | 1 | - | - | - | - | - | - | - | - | - | 3 |
| *Candida parapsilosis* | 1 | 2 | 1 | - | - | - | - | - | - | - | - | 4 |
| *Candida tropicalis* | - | - | - | 1 | - | - | - | - | - | - | - | 1 |
| *Cladosporium cladosporioides* | 4 | 6 | 18 | - | - | - | - | 1 | 1 | - | - | 30 |
| *Cladosporium sphaerospermum* | 1 | 1 | 2 | 3 | - | - | - | - | - | 1 | - | 8 |
| *Colletotrichum truncatum* | 1 | 1 | 1 | - | - | - | - | - | - | - | - | 3 |
| *Curvularia lunata* | 1 | - | - | - | - | - | - | - | - | - | - | 1 |
| *Cutaneotrichosporon cyanovorans* | - | 1 | - | 1 | - | - | - | - | 1 | - | - | 3 |
| *Cystofilobasidium macerans* | - | - | - | 1 | - | - | - | - | - | - | - | 1 |
| *Didymella glomerata* | 2 | 2 | 1 | 2 | - | - | - | - | - | - | - | 7 |
| *Exophiala equina* | 2 | 1 | 4 | 2 | - | - | - | - | - | - | - | 9 |
| *Exophiala oligosperma* | - | - | 1 | - | - | - | - | - | - | - | - | 1 |
| *Filobasidium magnum* | 1 | 2 | 1 | 3 | - | - | - | - | 2 | - | - | 9 |
| *Fusarium solani* | 3 | 4 | 3 | 1 | - | - | - | - | - | - | - | 11 |
| *Malassezia globosa* | 2 | - | 3 | - | - | - | - | - | 1 | 1 | - | 7 |
| *Malassezia restricta* | 13 | 10 | 17 | 11 | 1 | - | - | - | 4 | 5 | - | 61 |
| *Naganishia albida* | - | 1 | 1 | 3 | - | - | - | - | 1 | - | - | 6 |
| *Papiliotrema laurentii* | - | - | - | 1 | - | - | - | - | - | - | - | 1 |
| *Parengyodontium album* | - | 4 | 11 | 5 | - | - | - | - | 1 | - | - | 21 |
| *Penicillium citrinum* | 1 | 1 | - | - | - | - | - | - | - | - | - | 2 |
| *Penicillium decumbens* | - | - | 1 | - | - | - | - | - | - | - | - | 1 |
| *Penicillium oxalicum* | 1 | - | - | - | 1 | - | - | - | - | - | - | 2 |
| *Phlebia tremellosa* | - | - | 1 | - | - | - | - | - | - | - | - | 1 |
| *Pseudogymnoascus destructans* | 21 | - | 10 | 30 | - | - | - | - | 5 | 2 | - | 68 |
| *Purpureocillium lilacinum* | 1 | - | - | - | - | - | - | - | - | - | - | 1 |
| *Rhodotorula mucilaginosa* | - | 4 | 4 | - | - | - | - | - | 1 | - | - | 9 |
| *Sarocladium kiliense* | - | - | 1 | - | - | - | - | - | - | - | - | 1 |
| *Staphylotrichum coccosporum* | 1 | - | - | - | - | - | - | - | - | - | - | 1 |
| *Thermomyces lanuginosus* | 2 | 2 | - | 1 | - | - | - | - | - | - | - | 5 |
| *Trichoderma citrinoviride* | - | - | 1 | - | - | - | - | - | - | - | - | 1 |
| *Trichomonascus ciferrii* | - | 1 | - | - | - | - | - | - | - | - | - | 1 |
| *Wickerhamomyces anomalus* | 1 | - | - | - | - | - | - | - | - | - | - | 1 |

The number in columns indicate the number of samples in which fungal species was detected.

**
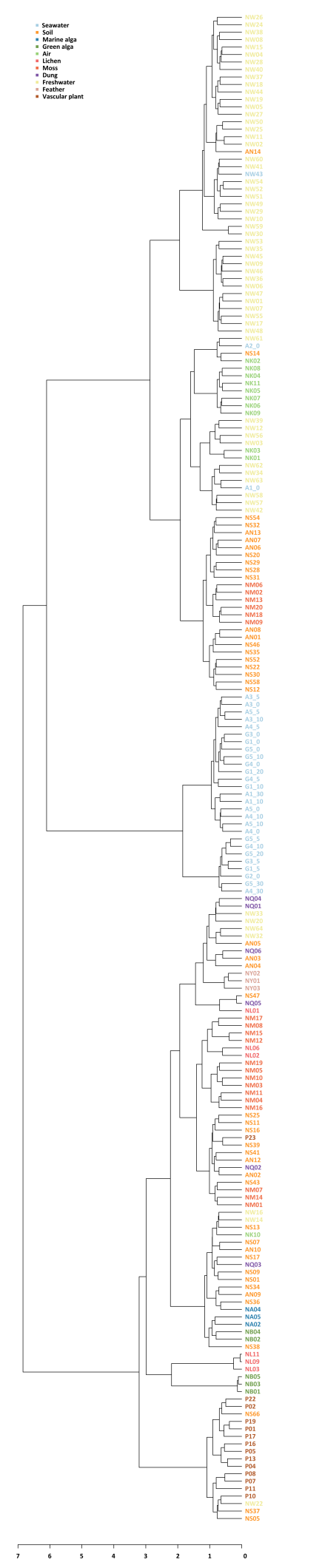
**

**Fig. S1.** Dendrogram showing fungal communities in the 202 samples of eleven habitats from the Fildes Region (maritime Antarctica).

**
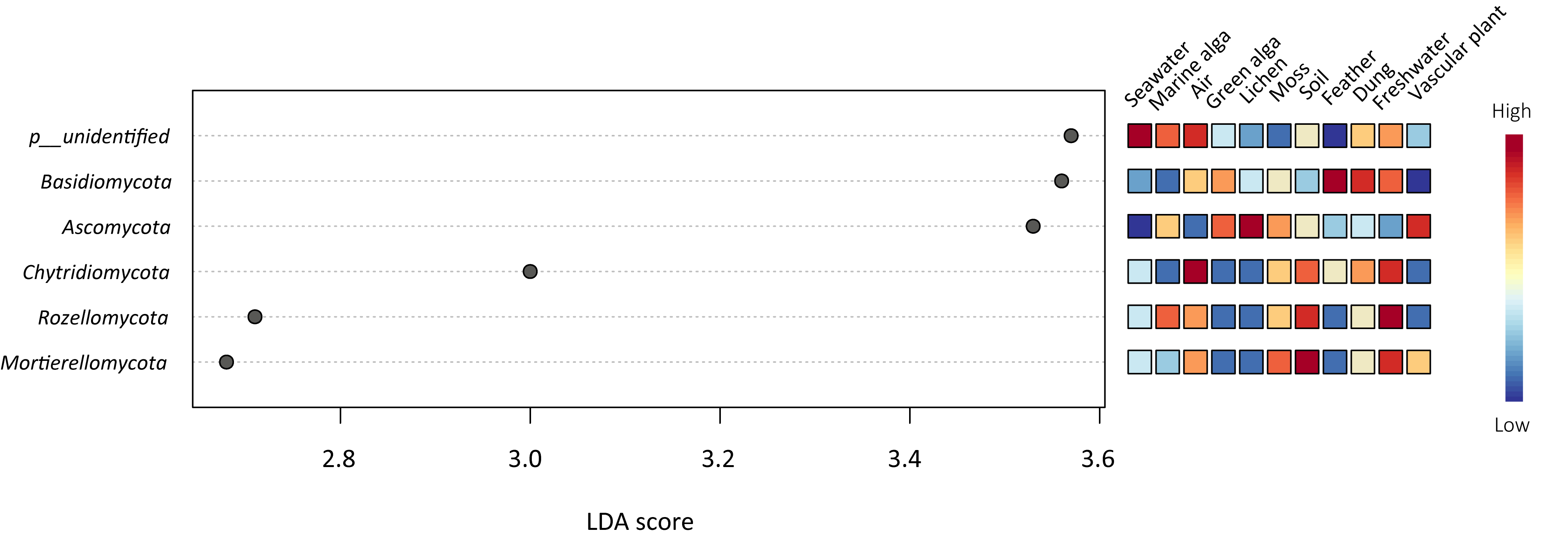
**

**Fig. S2.** LEfSe analysis showing the fungal phyla that are significantly different among the eleven habitats in the Fildes Region (maritime Antarctica). Significant phyla are ranked by their LDA scores (x-axis). The right heatmap shows whether the relative abundances of phyla are higher (red) or lower (blue).

**
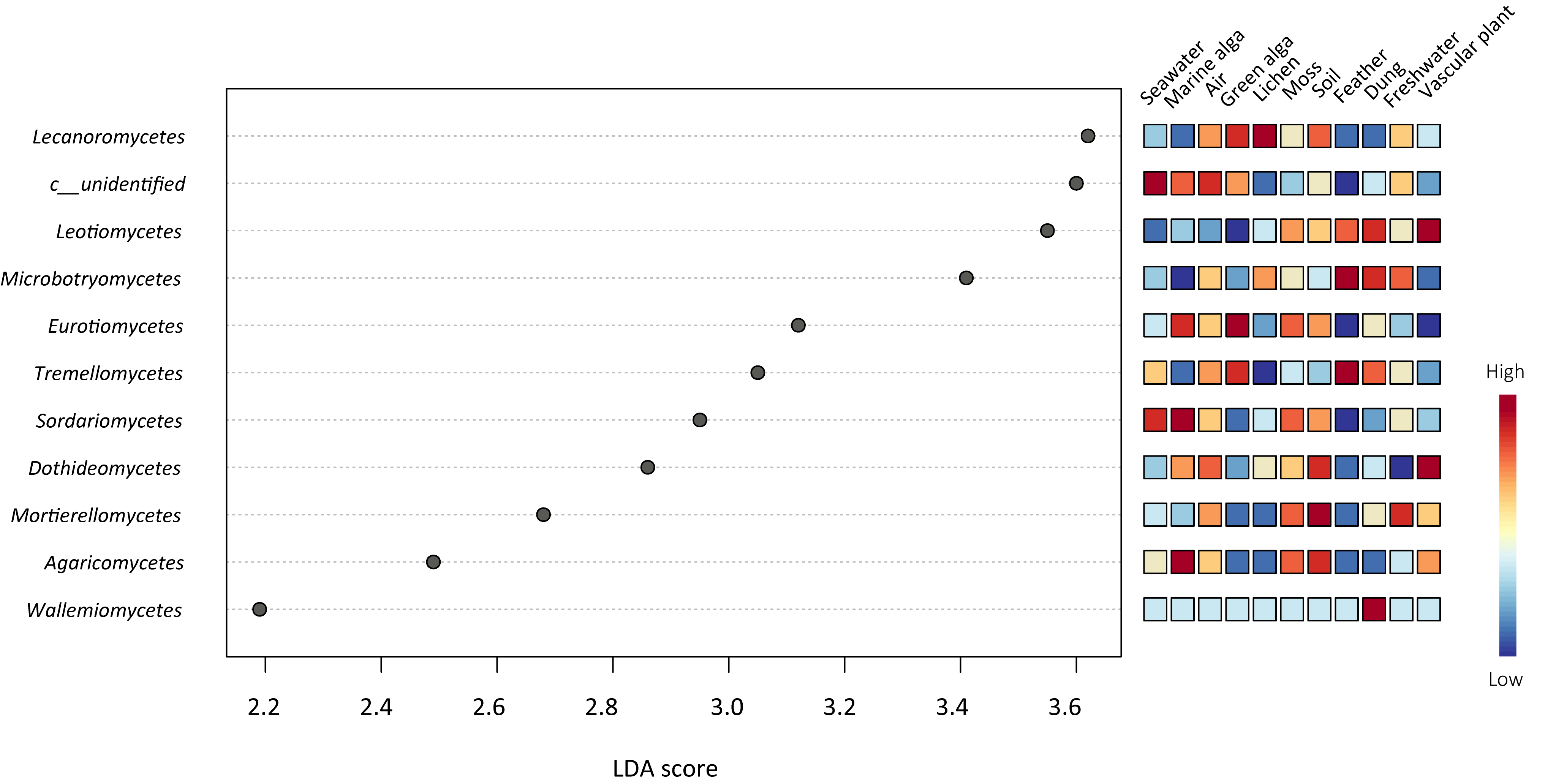
**

**Fig. S3.** LEfSe analysis showing the fungal classes that are significantly different among the eleven habitats in the Fildes Region (maritime Antarctica). Significant classes are ranked by their LDA scores (x-axis). The right heatmap shows whether the relative abundances of classes are higher (red) or lower (blue).

**
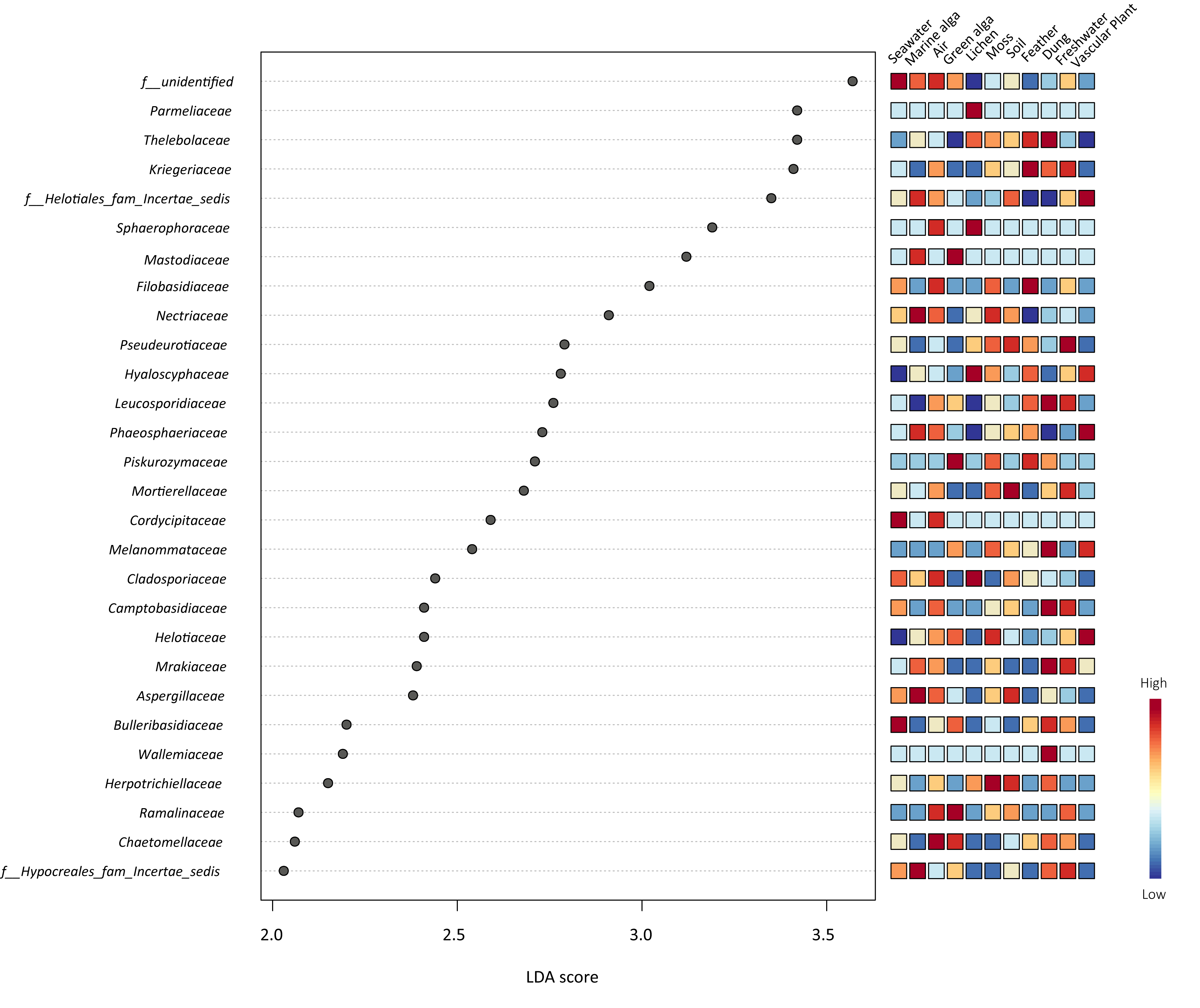
**

**Fig. S4.** LEfSe analysis showing the fungal families that are significantly different among the eleven habitats in the Fildes Region (maritime Antarctica). Significant families are ranked by their LDA scores (x-axis). The right heatmap shows whether the relative abundances of families are higher (red) or lower (blue).
